# The Graphic

**Published:** 1873-05

**Authors:** 


***The Graphic.***

There comes to the JOURNAL, under this title, a new daily evening paper from New York City with such remarkable peculiarities that we cannot think it out of place to notice it in our pages. It started out scarcely two months since, and has already achieved a success unparalleled in the history of journalism. Seven steam presses, aided by the most extensive machinery, have proved wholly inadequate to supply the daily demand. Three editions are issued each afternoon, containing not only all the current news, but three and a half pages of pictures illustrating all important events wherever they occur. By a new process, with the details of which we are not familiar, photography affords the type from which the imprint is immediately taken. Events of the last twenty-four hours are thus given in a style of illustration heretofore only attempted after a considerable period by ordinary illustrated papers, such as Harper's, Leslie's, etc. The further application of this wonderful art suggests itself in numberless ways. The vast expense attendant upon even the commonest engravings has heretofore prevented their coming into more than occasional use. Within a brief period we hope to see it brought into general use for the illustration of reports of surgical cases of all sorts, and even many varieties of so-called medical disorders, outlines of apparatus, etc., etc. The invention which prints, almost instantaneously, is certainly worthy of more than a passing notice.

Aside from its peculiar character as an illustrated paper we also find "The Graphic" in every sense a *news*paper, fully up to its most enterprising contemporaries, and its various editorial notes and reflections, keen and sensible, crisp and sparkling.

THE GRAPHIC, daily, is afforded at the extraordinarily low price of $12.00 per year. Single copies, 5 cents. Address THE GRAPHIC COMPANY, New York City.

